# A Novel Action of Endocrine-Disrupting Chemicals on Wildlife; DDT and Its Derivatives Have Remained in the Environment

**DOI:** 10.3390/ijms19051377

**Published:** 2018-05-05

**Authors:** Ayami Matsushima

**Affiliations:** Laboratory of Structure-Function Biochemistry, Department of Chemistry, Faculty of Science, Kyushu University, Fukuoka 819-0395, Japan; ayami@chem.kyushu-univ.jp; Tel.: +81-92-802-4159

**Keywords:** bisphenol A (BPA), bisphenol C (BPC), endocrine-disrupting chemical (EDC), dichlorodiphenyltrichloroethane (DDT), dichlorodiphenyldichloroethylene (DDE), 2,2-bis(*p*-hydroxyphenyl)-1,1,1-trichloroethane (HTPE), Lake Apopka, white rot fungi

## Abstract

Huge numbers of chemicals are released uncontrolled into the environment and some of these chemicals induce unwanted biological effects, both on wildlife and humans. One class of these chemicals are endocrine-disrupting chemicals (EDCs), which are released even though EDCs can affect not only the functions of steroid hormones but also of various signaling molecules, including any ligand-mediated signal transduction pathways. Dichlorodiphenyltrichloroethane (DDT), a pesticide that is already banned, is one of the best-publicized EDCs and its metabolites have been considered to cause adverse effects on wildlife, even though the exact molecular mechanisms of the abnormalities it causes still remain obscure. Recently, an industrial raw material, bisphenol A (BPA), has attracted worldwide attention as an EDC because it induces developmental abnormalities even at low-dose exposures. DDT and BPA derivatives have structural similarities in their chemical features. In this short review, unclear points on the molecular mechanisms of adverse effects of DDT found on alligators are summarized from data in the literature, and recent experimental and molecular research on BPA derivatives is investigated to introduce novel perspectives on BPA derivatives. Especially, a recently developed BPA derivative, bisphenol C (BPC), is structurally similar to a DDT derivative called dichlorodiphenyldichloroethylene (DDE).

## 1. Introduction

Numerous chemicals are utilized in our modern society; in some cases, such chemicals accidentally cause undesirable harmful effects on humans and our environments. One such chemical, 1,1′-(2,2,2-trichloroethylidene)bis(4-chlorobenzene) (DDT), is a pesticide that has been used worldwide since the 1940s. Paul Hermann Müller who was a Swiss chemist received the Nobel Prize in Physiology or Medicine for this discovery of the high efficiency of DDT as a contact poison against several arthropods in 1948. The use of DDT, however, was first banned in 1970 in Sweden based on many ecological considerations and the potential harm to human health, and subsequently in 1972 in the United States, followed by many other countries.

One of the well-known cases of environmental chemical contamination is in Lake Apopka in the state of Florida [1], which was caused by the spill of a pesticide mixture composed mainly of dicofol but also of DDT, DDD, and DDE in 1980. The decline in population levels and egg viability of alligators in Lake Apopka declined in the 1980s indicates that there seems to be some relationship between the 1980 pesticide spill and the acute reproductive failure. The most probable cause of these adverse effects is considered to be DDE, which acts as an antagonist to androgen receptor (AR) [2]; however, the exact molecular mechanisms underlying this phenomenon remain obscure.

Methoxychlor, [1,1,1-trichloro-2,2-bis(4-methoxy-phenyl)ethane], is another banned organochlorine insecticide effective against a wide range of pests [3]. The metabolite of methoxychlor, 2,2-bis(*p*-hydroxyphenyl)-1,1,1-trichloroethane (HTPE), is a strong binder of estrogen receptor α (ERα) with IC_50_ = 59.1 nM, and of ERβ with IC_50_ = 18.1 nM [4]. HPTE acts as an ERα agonist and an ERβ antagonist in the reporter gene assay using estrogen responsive elements in HeLa cells [5]. Monodehydroxy- and dihydroxy-DDE are also known to be the agonists for ERα and dihydroxy-DDE shows strong agonistic activity similar to HPTE [5]. The other well-recognized endocrine-disrupting chemical (EDC), Bisphenol A [2,2-bis(4-hydroxy-phenyl)propane (BPA)], which is used as a raw material in the production of polycarbonate plastics and epoxy resins all over the world, shows a considerably weak binding ability to both ERα and ERβ. However, bisphenol AF [1,1,1,3,3,3-hexafluoro-2,2-bis(4-hydroxy-phenyl)propane (BPAF)], which has a similar chemical structure to BPA, binds much more strongly to both ERα and ERβ than BPA does. BPAF is an analogous compound of BPA, in which all of the six hydrogen atoms at the two methyl groups in BPA are substituted by six fluorine atoms. BPA is able to activate both of ERα and ERβ considerably weakly in vitro in reporter gene assay, whereas, BPAF shows agonistic activity with ERα and distinct antagonistic activity with ERβ similar to HPTE. Notably, all of these compounds have mono- or di-phenyl groups.

The chemical structures of HTPE, BPA, and BPA derivatives are similar to those of DDT and its derivatives, in terms of possessing two benzene rings with some functional groups at their *para*-positions. The aim of this short review is to briefly summarize the discrepancy between the impact of EDCs, DDT, and its derivatives, on wild alligators in the Lake Apopka, and the results of laboratory experiments on their toxicological outcomes. The novel concept to fill in this discrepancy is proposed in the perspective of chemistry.

## 2. Continuing Obscurity of the Molecular Mechanisms of Adverse Effects in Alligators in Lake Apopka

The numbers of juvenile alligators in Lake Apopka in Florida severely declined in the 1980s and the main cause of this was considered to be reproductive failure [1]. Developmental abnormalities in gonads and abnormal sex hormone concentrations in juvenile alligators in the 1980s were precisely reported [6,7]. The widespread spill of a pesticide mixture mainly composed of dicofol—but also containing DDT; chloro-DDT; and metabolites of DDT, DDD, and DDE—in the 1980s [6] is considered to be the main cause of gonadal abnormality observed in wild alligators and the severe decline of their numbers. Some other EDCs have also been hypothesized to affect the organization of reproductive, immune, and nervous systems, especially during embryonic development in wildlife [8]. The smaller penis size and lower plasma testosterone concentration in juvenile alligators in Lake Apopka compared with other nearby lakes have been considered to be caused not by some estrogenic compounds but by the antiandrogenic effect of DDE [9]. 

The aforementioned accumulated results of scientific field research strongly indicated that some correlation between the spill of pesticides mixture in Lake Apopka and the gonadal and developmental abnormalities. To understand the molecular mechanism of these abnormalities observed in alligators, the expressions of the xenobiotic gene P450 and steroid hormone genes were measured [10,11], and the interactions between environmental chemicals, such as DDT, and the estrogen receptors and progesterone receptor were evaluated by using the extracts from the oviducts of alligators [12]. However, the actual chemical compounds and/or molecular mechanisms that were responsible for induction of the adverse effects on wild alligators still remain unknown. DDE is a well-studied organochlorine and is known to induce eggshell thinning and reproductive failure in birds, however, no significant correlation was observed between the mean thickness index of alligators′ eggs in Lake Apopka and those in the other clean lakes, for example, Lake Griffin [13]. The association between serum concentration of testosterone and/or 17β-estradiol and body size of alligators in Lake Apopka and those in the other clean lakes was also analyzed, but these relationships differed among the lakes; therefore, no obvious association or difference could be identified [14,15]. Several reports showed that the plasma testosterone concentration was to affect the phallus size of male alligators in Lake Apopka [16]; however, no correlation was found between the phallus size and serum concentrations of any specific contaminants measured, including DDT, DDE, and DDT [17]. DDE elicited antiandrogenic effects by interacting AR [2]; however, there is no direct evidence that DDE can decrease plasma testosterone concentration [6,8,10,15,16,17]. The whole genome sequences of three alligator species were submitted in 2012 [18], and it has only recently become possible to perform molecular studies on alligators, such as exhaustive sequence analyses of environmentally responsive genes [19]. As described in various research papers mentioned above, the exact molecular mechanisms underlying the adverse effects exerted by DDT and its derivatives on wildlife remains unclear.

## 3. Dichlorodiphenyltrichloroethane (DDT) Metabolites and Their Structurally Analogous Chemical Compounds Bisphenol C (BPC)

DDT is an already banned pesticide and its well-publicized researches are reviewed by the International Program on Chemical Safety (IPCS), which is a collaborative program of the United Nations Environment Program (UNEP), the International Labor Organization (ILO), and the World Health Organization (WHO) [20,21]. DDT is also referred to as *p*,*p*′-DDT, and the commercially used pesticide products contained its accompanied substitution isomer 2-(2-chlorophenyl)-2-(4-chlorophenyl)-1,1,1-trichloroethane (*p*,*p*′-DDT). A number of studies on DDT and on its metabolites have been published to date, including the detailed isomeric composition of DDT in the environment [22], a brief history of the usage of DDT [23], reductive metabolism of both *p*,*p*′-DDT and *p*,*p*′-DDT [24], and biomonitoring equivalents for DDT [25]. Bioaccumulations of DDT have been reported in fish [26], in birds [27], and also in humans [28]. Particularly, DDE is one of metabolites of DDT, and it is considered to be the main causes of adverse effects in animals. It is natural to consider that aquatic animals are easily affected by EDCs; thus, various effects of DDE exposure are analyzed using fish [26,27,29,30]. However, few studies have focused on mammals as well. A strong positive association between breast cancer risk and adipose or blood concentrations of DDT has previously been reported by some experimental results and a cohort study; however, it was concluded that the relationship between them remains unclear because the association was dependent on the area or race [31]. 

DDT rarely binds to sex hormone receptors [2], and thus, one of the DDT metabolites in the body, DDE, is considered to be the EDC to induce the adverse effects on wildlife, especially on male alligators in Lake Apopka [9]. However, the binding abilities of DDT and DDE in vitro are considerably weaker for sex hormone receptors. The most potent binder of the androgen receptor among the known DDT metabolites, *p*,*p*′-DDE, showed the 50% inhibition concentration value (IC_50_) of 5,000 nM by competitive binding assay using rat ventral prostate extracts, whereas these compounds and DDT hardly bind to the estrogen receptors in the uterine cytosolic extract (IC_50_ value of >1,000,000 nM) [2]. The intrinsic estrogen, 17β-estradiol (E2), had the IC_50_ value of 2 nM against the estrogen receptors in the same uterine cytosolic extract [2]. It had been reported that the concentration of *p*,*p*′-DDE in alligator eggs from Lake Apopka in 1984 and in 1985 were 5.8 ppm and 3.5 ppm, respectively. These concentrations corresponded to ca. 18,000 nM for 5.8 ppm and ca. 11,000 nM for 3.5 ppm of *p*,*p*′-DDE. It, therefore, seemed reasonable to assume that *p*,*p*′-DDE disrupted the endocrine systems controlled by androgens. However, it was not apparent whether *p*,*p*′-DDE exposure lead to the reduced hatching success of eggs in Lake Apopka or not, because the *p*,*p*′-DDE concentration in the sample egg and hatching success of the remaining eggs in the clutch showed no significant negative correlation [13]. 

**Figure 1:**
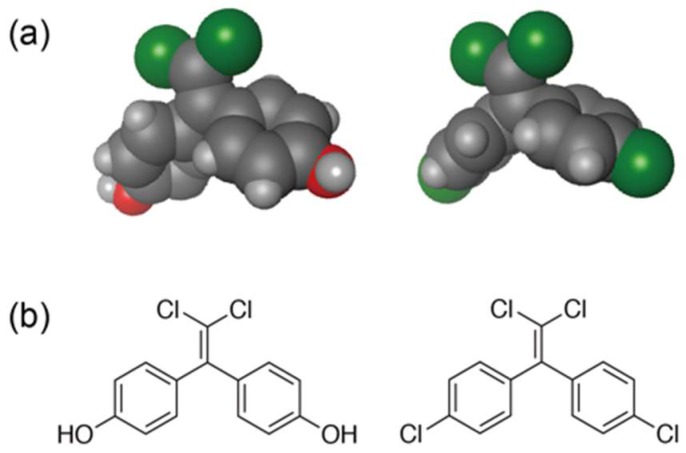
Structures of *p*,*p*′-DDE and BPC. (**a**) CPK models of *p*,*p*′-DDE (**right**) and BPC (**left**), (**b**) chemical structures of *p*,*p*′-DDE (**right**) and BPC (**left**). The difference between *p*,*p*′-DDE and BPC is only two functional groups at each *para*-position of the benzene ring.

The *p*,*p*′-DDE possesses four chlorine atoms, and two of them are covalently bound to benzene rings. It should be noted that one of the bisphenol A (BPA) derivatives, bisphenol C (BPC), has a similar chemical structure to *p*,*p*′-DDE (Figure 1). The BPA is an endocrine-disrupting chemical used as a raw material of polycarbonate plastics and epoxy resins [32,33,34,35,36,37] and BPA is definitely detected in human serum and urine [38,39,40]. The concern for the effects of BPA on the brain, behavior, and prostate gland of human fetuses and children has been declared by The National Toxicology Program (NTP) Center for Evaluation of Risk to Human Reproduction (CERHR) of the US National Institutes of Health, even at present levels of BPA exposure to humans [41], while some controversial topics on the NTP-CERHR monograph have also been reported [42,43,44]. BPA was initially been considered to induce adverse effects by interacting with estrogen receptor [45,46,47], and various lines of research have also been reported on the molecular mechanisms of its adverse effects, such as epigenetic effects of BPA exposure and the development of cancer [48,49,50,51,52]. The unusual aspect of BPA is that even “low doses” of BPA have been correlated with adverse effects on experimental animals [53,54,55,56]. However, the molecular mechanism of “low-dose effects” induced by BPA has been remains poorly understood, because BPA is a considerably weak binder to estrogen receptors. It was recently reported that BPA strongly binds to the other nuclear receptors, estrogen-related receptor γ (ERRγ) [57,58,59,60] and the constitutive androstane receptor (CAR) [61], but BPA does not influence the high constitutive transcriptional activities of both the nuclear receptors. In order to summarize the full range of latent health effects of BPA exposure and to provide data to be used for regulatory decisions, the collaborative research program known as the Consortium Linking Academic and Regulatory Insights on BPA Toxicology (CLARITY-BPA) has been developed by the National Toxicology Program (NTP), National Institute of Environmental Health Sciences (NIEHS), and U.S. Food and Drug Administration (FDA), and is currently in progress with the aim of declaring the final CLARITY-BPA conclusion by August 2019 [62,63].

To date, various BPA derivatives are utilized as raw materials of highly functional plastics and as alternatives of BPA. Numerous studies have been conducted on BPA, mainly with the point of view of safety, while few studies have analyzed the potential health risks of BPA derivatives [64]. One of BPA the derivatives, BPC, was first reported in a patent in 1964 [65] and a brief paper regarding BPC was published in 1968 [66] as a monomer for polycarbonates, and the physical properties of the BPC homopolymer have been summarized focusing on its highly flame-resistant properties [4,67,68,69,70,71]. Recent research indicated that BPC is definitely released into the environment, for example, as wastewater in lakes, and is also found non-human milk samples [72,73,74]. BPC has been reported to possess the ability to influence the transcriptional activity of ERα and ERβ [75,76]; some computer simulation studies were applied to afford the structural basis of these biochemical observations [77,78]. The X-ray crystal structural analysis of the ERα/BPC complex has illustrated that BPC bind to the ligand binding pocket of ERα where actually the natural ligand E2 binds [75]. Notably, halogen-containing bisphenol derivatives such as BPC and bisphenol AF (BPAF) also showed agonistic activity for ERα but antagonistic activity for ERβ [4,75,79]. We previously confirmed that BPC is a strong binder both for ERα and ERβ with IC_50_ values of ca. 3 nM. A similar observation of this distinctive feature has also been reported by using HPTE [1,1,1-trichloro-2,2-bis(4-hydroxyphenyl)ethane], a metabolite of the already banned pesticide methoxychlor [1,1,1-trichloro-2,2-bis(4-methoxyphenyl)ethane] [5]. Human hepatoma cells (HepG2) and HeLa cells were transiently transfected with either human or rat ERα or ERβ, and the transcriptional activities of E2 and HPTE were analyzed. HPTE behaved as an ERα agonist and an ERβ antagonist with estrogen-responsive promoters. To date, various lines of experiments have been performed to analyze the adverse effects of HPTE. HPTE induced the reduction of testosterone production in rat Leydig cells [80], caused the difference in the gene expression patterns in mice reproductive organs as uterine and ovarian tissues [81,82,83], caused inhibition of cAMP production in rat granulosa cells [84], caused inhibition of one of the cytochrome P450 (CYP) enzymes CYP11A1 [85,86] and 3β-hydroxysteroid dehydrogenase [87], and caused the activation of some kinases such as mitogen-activated protein kinase (MAPK) and phosphatidylinositol-3-kinase [88]. However, it has not been reported whether BPC possesses similar properties to HPTE regarding the activation or inhibition of these intrinsic enzymes in vivo and in vitro.

## 4. Halogen Bonds in Biological Molecules 

Above mentioned all of EDCs; HPTE, BPAF, and BPC, are small and rigid chemical compounds containing halogen atoms, which have molecular weight of around 300. The natural thyroid hormones thyroxine (T4) [3,5,3′,5′-tetraiodothyronine] and 3,5,3′-triiodothyronine (T3) have several iodine atoms in their chemical structures as well [89]; however, generally halogen atoms are rarely found in intrinsic biological molecules. Regarding artificially produced chemicals, polychlorinated biphenyls (PCBs), which are already banned industrial materials [90,91], and tetrachlorodibenzo-*p*-dioxins (TCDDs), in which the most toxic form is 2,3,7,8-TCDD [92,93], contain several chlorine atoms. These halogen atoms facilitate the halogen bonds between ligand/receptor interaction [94,95,96]. The halogen bonds in biomolecular systems have received considerable attention and have been analyzed recently with the aim of more efficient drug discovery [89,97], although it is a well-studied and a rather established interaction in the field of chemistry. Halogen bonds, also called halogen interactions, are defined by International Union of Pure and Applied Chemistry (IUPAC) as “A halogen bond occurs” when there is evidence of a net attractive interaction between an electrophilic region associated with a halogen atom in a molecular entity and a nucleophilic region in another, or the same, molecular entity [98].” The heavier halogen atoms increase the strength of halogen bonds in the order of Cl < Br < I, and halogen bonds with fluorine atoms are rarely found. This tendency can be explained by the increasing polarizability from F toward I in this periodic group [99]. The halogen bond between phenyl-X and O=C has been estimated to contribute to a gain in free enthalpy of −ΔΔG = 2.6 kcal·mol^−1^ [100,101], and these relatively weak interactions are useful for the improvement of drug–target binding affinity [102]. Various computational calculation analyses have been performed to evaluate and elucidate the halogen bonds between receptor proteins and ligand chemicals [102,103] containing iodine atoms [104], chlorine atoms [105], and bromine atoms [106]. Halogen bonds are one of the defining non-covalent bonds resulting in electrophilic region on the top of halogen atoms, which is known as the “σ-hole”. Thus, halogen bonds are also called as σ-hole bonds or σ-hole interactions [94], and their counterparts are electron-rich atoms or functional groups known as π-systems. Oxygen atoms of peptide bonds in heavy chains and π-systems of aromatic groups in side chains of tyrosines, phenylalanines, histidines, and tryptophans are appropriate interacting partners for σ-holes. Halogen bonds are utilized not only in the interactions between receptor proteins and ligand chemicals but also in the modulation and stabilization of short peptides and proteins intramolecularly [105,106]. In a previous study, a peptide comprising 10 amino acid residues was designed to form a β-hairpin in solution, which had a chlorine atom to introduce a halogen bond. The introduced halogen bond stabilized the β-hairpin structure [105]. As for the protein structure, noncanonical amino acids, such as halogen-containing phenylalanines, were directly introduced to T4 lysozyme at the position of tyrosine by using AMBER (TAG) codon, and the obtained constructs were expressed in E. coli and purified. No halogen-introduced T4 lysozyme was stabilized compared to the wild type presumably due to thermal melting points; however, the paper provided a new concept for stabilization of the protein by engineered halogen bonds [106].

## 5. The Possibility of the Conversion from DDT to BPC

**Figure 2:**
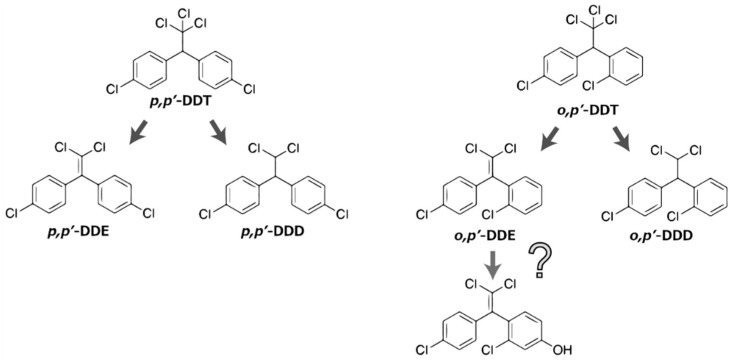
Structures of DDT and its metabolites. DDT used as a pesticide is a mixture of its isomers. *o*,*p*′-DDE is to be a potential chemical compound for endocrine disruption in wildlife in case it is hydroxylated at the *para*-position.

Is it possible to convert DDT to BPC directly? The chemical structure of *p*,*p*′-DDE, a *p*,*p*′-DDT metabolite, is quite similar to BPC, in which two of the chlorine atoms of *p*,*p*′-DDE were substituted to the two of hydroxyl groups. However, in the point of view of chemistry, it is hard to proceed with the direct replacement reaction of chlorine atoms connected at the *para*-position of the benzene ring of *p*,*p*′-DDE to hydroxyl groups found in the chemical structure of BPC. As shown by the crystal structure analyses, at least one hydroxyl group connected to *para*-position of a benzene ring is essential for ligand binding to ERα [107] and ERβ at their specific ligand binding regions [108,109], and hydroxyl groups of the natural ligand dihydrotestosterone interacts with the androgen receptor [110]. The new pathway is required to be transformed from DDT to BPC or BPC derivatives, which have at least one hydroxyl group at the *para*-position of the benzene ring. The term DDT generally refers to *p*,*p*′-DDT, but technically DDT used as a pesticide is a mixture of its isomers and had the following constituents: *p*,*p*′-DDT, 77.1%; *o*,*p*′-DDT, 14.9%; *p*,*p*′-TDE [1,1′-(2,2-dichloroethylidene)-bis(4-chlorobenzene)], 0.3%; *o*,*p*′-TDE, 0.1%; *p*,*p*′-DDE, 4%; *o*,*p*′-DDE, 0.1%; and unidentified products, 3.5% [20,21]. This indicates that a considerable amount of *o*,*p*′-DDT is transformed to *o*,*p*′-DDE (Figure 2). Halogen atoms are rich in electrons, and halogen atoms at the *para*-position activated the reactivity of hydrogen atoms at the *ortho*- and meta-positions of the same benzene ring. This seems to be an important aspect with regard to consider the conversion of DDT derivatives to BPC derivatives, because according to this concept, there is sufficient potential to convert a hydrogen atom to a hydroxyl group at the *para*-position of the benzene ring adjacent to the chlorine atom at its *ortho*-position.

The chemicals emitted into the environment are to be subjected to the various kinds of microorganisms, and to be broken down in many cases. It has been discovered that several strains of white rot fungi are able to degrade DDT, PCBs, and dioxins in soil [111,112,113,114,115,116,117]. White rot fungi are able to break down the lignins which are rigid and complex phenolic polymers found in cell walls of wood. DDT, PCBs, and dioxins have roughly similar chemical structures to the typical monomers composing lignin polymers that have the benzene ring with monolignols. It was reported that the transformation pathway of DDT was dependent on species of fungi, because the different metabolic products were detected on laboratory experiments using HPLC and gas chromatography-mass spectrometry (GC/MS) analyses [116]. Some types of white rot fungi, which are able to degrade DDT, produce some cytochrome P450 enzymes, and these enzymes are involved in the hydroxylation of organic xenobiotic chemicals [118,119]. Currently, there is no report on the identification of BPC or related compounds as the degraded product of *p*,*p*′-DDE; however, chlorine atoms on the benzene ring enhanced the reactivity of contiguous hydrogen atoms; thus, the *para*-position of *p*,*p*′-DDE has a high possibility of replacement with a hydroxyl group by some enzymes in fungi. This reaction leads to the production of chemicals similar to BPC. Further research is needed to identify the BPC-related degraded products and some novel enzymes to afford this reaction.

## 6. Conclusions and Future Perspectives

DDT was previously a widely used chemical insecticide that is now recognized as a persistent organic pollutant (POP) and is banned in many countries. However, this chemical is still used for the malaria vector control in some areas, especially recommended for indoor residual spraying by World Health Organization (WHO) [120], simply because there is no cost-effective and equivalently efficient pesticide to be used as an alternative to DDT. DDT is now recommended to be used under well-established and effective protocols; however, it is still detected from the serum in some contaminated areas [28]. DDT and its metabolites have affected the wildlife, for example, DDE, which is one of the DDT metabolites, induced thinly-shelled eggs in birds; however, the exact mechanism of this eggshell thinning remains unknown [21]. DDT and its metabolites have similar chemical structures to BPA derivatives, which are a widely utilized raw material of polycarbonate plastics and epoxy resins. Remarkably, the difference between *p*,*p*′-DDE and BPC is due to only two functional groups at each *para*-position of the benzene ring; chlorine atoms in *p*,*p*′-DDE and hydroxyl groups in BPC. This structural feature has been the focus of research since 2002 [121]; nevertheless, the chemical characteristics of a chlorine atom and a hydroxyl group are quite different from each other. While an estrogenic chemical *o*,*p*′-DDT causes intersexuality in fish [29], X-ray crystal structures of ligand-bound ERs showed that the importance of hydrogen bonds between a hydroxyl group in ligands and a glutamic acid and/or an arginine in ERs, respectively [75,107,122]. However, it is difficult to directly replace a chlorine atom of the benzene ring with a hydroxyl group from the point of view of chemistry. In this review, a novel concept has been proposed that *o*,*p*′-DDE, which is derived from the residual product of *p*,*p*′-DDT, could be the causal agent for endocrine disruption in wildlife generated by hydroxylation at its vacant *para* position by enzymes released by some microorganisms in soil. White rot fungi, which have already been utilized in bioremediation, are potent candidates for generation of the enzyme that can produce BPC derivatives from *o*,*p*′-DDE. The exact molecular mechanisms for the conversion of *o*,*p*′-DDT to BPC derivatives need to be identified in future research analyses.

## Abbreviations

BPA	Bisphenol A
BPC	Bisphenol C
EDC	Endocrine-disrupting chemical
DDT	Dichlorodiphenyltrichloroethane
DDE	Dichlorodiphenyldichloroethylene
ER	Estrogen receptor
HTPE	2,2-bis(*p*-hydroxyphenyl)-1,1,1-trichloroethane
